# Heart Rate Variability Analysis in Revascularized Individuals Submitted to an Anaerobic Potency Test

**Published:** 2007-10-22

**Authors:** Geraldo Mendes Gutian, Leandro Yukio Alves Kawaguchi, Alessandra de Almeida Fagundes, Adriana Kowalesky Russo, Emmelin Souza Monteiro, Andrea Monteiro, Alderico R de Paula, Wellington Ribeiro, Rodrigo Alexis Lazo Osorio

**Affiliations:** 1 Laboratorio de Reabilitacao Cardiovascular da Universidade do Vale do Paraiba (Univap) (Laboratory of Cardiovascular Rehabilitation of the University of the Vale do Paraiba); 2 Instituto de Pesquisa e Desenvolvimento (IP&D da Univap) Institute of Research and Development (IP&D - Univap); 3 Academia Militar das Agulhas Negras (AMAN) (Agulhas Negras Military Academy); 4 Universidade Federal de Sao Paulo Escola Paulista de Medicina (UNIFESP-EPM)

**Keywords:** Autonomic modulation, Modified Wingate Test, Myocardial revascularization, Heart Rate variability

## Abstract

The objective of this study was to analyze the behavior of autonomic modulation before, during and after the Modified Wingate Test (WanMT), through the analysis of Heart Rate Variability (HRV). Six volunteers between the ages of 40 and 70, post-revascularization procedures (angioplasty and/or surgery, mean duration 10 months), were submitted to supervised training for at least 10 to 14 months. The following protocol, divided into 5 phases, was used: 1) Rest Phase (RP): 180 seconds; 2) Submaximum Phase (SP): 30 seconds; 3) Maximum Phase (MP): 30 seconds; 4) Active Recuperation Phase (ARP); 120 seconds and; 5) Passive Recuperation Phase (PRP): 180 seconds. For the WanMT Test, we selected the load of 3.75% of corporal weight for all volunteers. To analyze the HRV, we used the following parameters: the interval RRr, MNN, SDNN, RMSSD and PNN50. We only observed results for the group according to RMSSD parameters during the rest phase of the test protocol in which the group remained in vagal presence and during all other phases in vagal depression. However, when we analyzed the PNN50, we observed that the group was in medium vagal presence during all of the phases of the test though there was no statistically significant difference (p> 0.05) between the phases. Therefore, we can say that all of the individuals had a similar profile in the autonomic response to the WanMT, confirmed by the parameters studied in the analysis of the HRV in the time domain.

## Introduction

The analysis of Heart Rate Variability (HRV) has become an extensively utilized noninvasive tool in the evaluation of cardiovascular autonomic nervous system functioning in various physiological situations [1-4]. The analysis of HRV in revascularized individuals has its importance since it can be used as a predictor of the evolution of cardiac disease, increasing the life expectancy of the population [5]. There are few studies involving HRV analysis during anaerobic exercise and the posterior behavior of the autonomic nervous system and its responses during and after physical activity. During daily activities, we observed some activities of a more intense character lasting for just a few seconds, characterizing predominantly anaerobic exercises. In this context, the study of responses to physical exercise is particularly useful, permitting an application of different levels of stress, quantifiable through the workload or the repercussions in the metabolic responses [6]. The variations in the duration of RR intervals depend on the activity of the sympathetic and parasympathetic nervous systems. These variations constitute what is commonly called Heart Rate variability (HRV). Its study permits us to recognize and characterize some situations in which the disease affects the autonomic control of the heart [5,7].

The objective of this study was to analyze the behavior of the autonomic modulation in revascularized individuals during and after the Modified Wingate Test (WanMT) through the analysis of the HRV in the time domain.

## Materials and Methods

### Casuistics

 The sample consisted of 6 males between the ages of 40 and 70. six post-revascularization procedures (two patients were post angioplasty and bypass surgery, two patients post angioplasty and two patients post bypass surgery). The patients were being treated with beta-blockers, vasodilators, diuretics, antiplatelet drugs, lipid-lowering drugs and oral antidiabetic drugs. Echocardiographic studies weren't done to evaluate the left ventricular function. All  the participants belonging to the Univap Cardiovascular Rehabilitation Program, were submitted to aerobic training. The six individuals received a well-elaborated explanation of the procedures and objectives that would be developed during the work. The participants also signed an individual "Free Informed Term of Consent" in which they were informed of the procedures and risks during the tests.

### Methods

The volunteers, who had at least 10 months of aerobic training, were submitted to a clinical evaluation. They were also oriented 24 hours before the Modified Wingate Tests to avoid any alternative activities during physical effort. The criteria for exclusion from this study were: diabetic neuropathy, atrial fibrillation, frequent atrial and ventricular arrhythmias, severe arterial hypertension [8] and Chagas disease.

 1- Physical Training: the volunteers had been training from 10 to 14 months at a load of 55 to 65% of functional capacity, 3 times a week, for a period of 50 minutes.

 2- Modified Wingate Test: this test was utilized for the determination of maximum anaerobic potency in the CYBEX cycloergometry. The test consisted of 30 seconds of exercise at maximum speed with a constant resistance equivalent to 3.75% of corporal weight [7].

 3- Material utilized for gathering electrocardiogram data: to collect  the Heart Rate Variability (HRV) data, we used an Extenser Pentium-2 Notebook with an analogical-digital DATAQ DI-194RS and ACTIVE ECAFIX monitor. The electrocardiographic register system chosen for this test was the CM5 Derivation, in accordance with the 1995 National Consensus on Ergometrics [9]. For the interpretation on the HRV data, we used the programs Matlab 4.0 and ANAVC.

4- The protocol used to analyze the Wingate Test HRV was divided into 5 phases. Rest Phase: duration of 180 seconds; Submaximum Phase: duration of 30 seconds; Maximum Phase: duration of 30 seconds; Active Recuperation Phase: duration of 120 seconds and Passive Recuperation Phase: duration of 180 seconds.

5- Parameters used to analyze the HRV: only parameters in the time domain were used: RR (NN) interval, SDNN (iRR standard deviation), PNN50 (percentage between the iRRs every 50 minutes) and RMSSD (root mean square of successive differences between iRRs).

6- Statistic Method: We employed statistics in accordance with the orientations of the Statistics Department of the Agulhas Negras Military Academy. Descriptive Statistics were used for age, weight, height, arterial pressure, Heart Rate at rest, time of physical training and use of medications. During the Wingate Test, the Student's Test and the Kruskal-Wallis Test were used for the comparison between physical and autonomic performance of each volunteer.

The statistic programs used were MINITAB 13.0 and STATISTIKA for comparison of the variables.

### Results

Table 1 presents the volunteers' average (X) and the standard deviations (SD) (n=6), data referring to age (years), stature (m), weight (kg), systolic and diastolic arterial pressure (in mmHg) and values of the maximum and absolute average strength (W), maximum and relative average strength (W.Kg), with their respective averages and standard deviations. During the Modified Wingate Test, one of the volunteers (**6**) presented the greatest maximum and average absolute strength and the greatest maximum and average relative strength (335 W.Kg^-1^, 274 W.Kg^-1^, 4.9 W.Kg^-1^ and 4 W.Kg^-1^), respectively, while two other volunteers (1 and 2) presented the smallest absolute maximum strength with 192 W.Kg^-1^ and the smallest relative maximum strength with 3.4 W.Kg^-1^.

The results observed in Table 2 show that four individuals were being treated with beta-blockers. The data on percentages of HR (heart rate) in the test indicate that four of the individuals had a HR of 80 to 90% of the maximum HR and two individuals had different HR; one of them had a HR of 66% and the other had a HR of 110% of the maximum HR. In relation to the percentage of recuperation, we can observe that four of the individuals attained recuperation between 66 and 87% and two individuals had a different recuperation; one did not recuperate (35%) and the other had a recuperation of 112.5% of HR at rest. Analyzing the results of the rate of fatigue in the test, we observed that the group's average was 34.1%, presenting good performance during the test, within the percentage of individual maximum intensity; one individual had a rate of fatigue of 69%, characterizing a drop in performance during the test.

The results show the individual performance of each volunteer during the test, indicating the values of the parameters in each phase, according to Table 3,4,5. Using the Kruskal-Wallis Test in the protocol phases, there was a significant difference among the group of volunteers (p<0.01) in the MNN and SDNN parameters (p>0.05), that is, the rejection of a null hypothesis (Ho) occurred among the volunteers. However, statistically, there was no significant difference in the RMSSD and PNN50 (p>0.05) parameters in the protocol phases.

In Table 4, we can observe the group's average according to the RMSSD parameter. The group continued to show vagal presence only in the rest phase and, in the other phases, we observed a decrease in vagal activity.

According to the TASK FORCE [6] rates in relation to RMSSD (in milliseconds), rates < 30 ms indicate vagal depression and rates >30 ms indicate vagal presence. In Table 5, we see the PNN50 parameter and can observe that the group's average had medium vagal presence during all of the protocol's phases. However, there was no statistically significant difference (p> 0.05) among the phases. When we observed the individual response of the volunteers, we noted that one of them (2) remained in vagal depression during all of the phases.

According to Kleiger et al. [10] the presence of vagal activity rates of < 4% indicate depression of vagal activity and rates between 4 and 24% indicate average vagal presence and > 24% indicate vagal presence.

## Discussion

### Wingate Test, Exercise Intensity and Heart Rate Variability (HRV)

Beneficial effects of physical training have been reported for post-myocardium infarct patients [11,12] and for patients after cardiac transplant [13]. In our study, when we analyzed the data on percentages of exercise intensity reached in the test. This indicated that four of the individuals had a submaximum intensity of 80 to 90% of the maximum test intensity and two individuals had different intensities. One of them reached a percentage of 66% and the other 110% of the maximum test intensity. In relation to the strengths, both relative and absolute, the values found in the study were smaller than those observed by Gordon et al., (1987) [14], testing volunteers with coronary diseases.

Recuperation of the HR autonomic regulation has been proposed for short terms, within minutes after the maximum and submaximum exercises [15]. In this way, slow HR recuperation after dynamic maximum and submaximum exercise in a short time is considered a powerful predictor of global mortality based on populational data [10]. In our study, we observed that four of the individuals achieved recuperation between 66 and 112.5% and one of the volunteers had a recuperation of 35% in relation to HR at rest. Analyzing the results of the rate of fatigue in the test, we observed that the group's average was 34.1%, presenting good performance during the test, within the percentage of individual maximum intensity, which was also observed by Gordon et al. [14]

### Rehabilitation and Analysis of Heart Rate Variability

Malfatto et al. [11] observed the effects of long and short-term physical training performed with patients who had had an infarct of the myocardium, and were submitted to 8 weeks of training. When comparing the pre- and post-training parameters, they arrived at the following conclusion: the SDNN parameter increased 25% (p>0.001); the PNN50 increased 120% (p>0.01) and the RMSSD increased 69% (p>0.01). However, after an 8-week cardiac rehabilitation program to which 14 elderly (73.9 ± 3.5 years) patients were submitted, Perini et al observed that there were no significant differences in the PNN50 and RMSSD (p>0.01), before and after the rehabilitation program.

On the other hand, for patients who had suffered acute myocardial infarction, the regular practice of physical exercise improved their functional capacity and actuated favorably on various factors of coronary [16]. In addition, it modified the autonomic cardiac activity, leading to a greater parasympathetic predominance, demonstrated experimentally in dogs [16,17] and in patients who had had recent infarcts [14].

Although a variable individual susceptibility exists for the magnitude of the effect of training on the parasympathetic function, this vagomimetic effect seems to construct one of the mechanisms through which physical training reduces the morbimortality after acute myocardial infarction. According to the rates of Kleiger et al. [10], in our study we observed that, according to the RMSSD parameter, the group remained in vagal presence only in the test protocol's rest phase; in the other phases, we observed a decrease of vagal activity. When we analyzed the PNN50 parameter, we noted that the group's average remained in medium vagal presence during all of the phases of the protocol. However, there was no statistically significant difference (p>0.05) among the phases. When we observed the volunteers' individual response, we also saw that one of the volunteers (**2**) had remained in vagal depression during all of the phases.

### Other Associated Factors and HRV

In our study, the volunteers were under pharmacological treatment. Of the four volunteers using beta-blockers (atenolol) during the cardiac rehabilitation program, three of them were using atenolol (25mg/day). Only one volunteer showed an increase in vagal tonus during the test. This could possibly be due to the use of beta-blockers (atenolol 50mg/day) [12,16,18].

In agreement with some authors, we concluded that the beta-blocker increased vagal tonus due to a sympathetic block and possibly to a mechanism of central action which is responsible for its protective effects in the post-AMI state [16]. Therefore, in athletes trained for resistance, the low resting heart rates are due to a reduction of the intrinsic heart frequency and not to an increase of parasympathetic tonus. This has been seen by using pharmacological blockers (propanolol and atropinas) to suppress the sympathetic and parasympathetic activity of the SNA [19]. Furthermore, Bonaduce et al. [20] arrived at the conclusion that mechanisms other than the changes in autonomic cardiac control could be involved in determining the deep bradycardia in athletes.

Regarding other risk factors, we can observe that four of the volunteers  had systemic arterial hypertension. However, this had no influence on the HRV observed in the results obtained in the D.T. On the other hand, two of the volunteers were diabetics and this constituted a second risk factor. In addition, two volunteers had hypercholesterolemia, which was part of their past pathological history. Another two volunteers presented a reduction of the parasympathetic during all phases of the test protocol, possibly due to these associated factors in the response to the Modified Wingate Test. Both diabetes and hypertension are factors that could have influence on the HRV due to an increase in the sympathetic tonus [6,19,19,21]

Considerably more research is needed to understand  the effects and clinical relevance of altered vagotonic and adrenergic tone on total HRV power and its various components in health and disease.

## Conclusion

1- The exercise intensity reached in the test indicates that four of the volunteers had a submaximum HR of 80 to 90% of the test's maximum intensity and two others had different HR. One had a percentage of 66% of the maximum HR and the other, 110% of the maximum HR for his age.

2- The volunteers were considered as trained due to their rapid recuperation after intense exercise activity. We observed that four of the individuals attained recuperation between 66 and 112.5% and another attained recuperation (35%) in relation to HR at rest.

3- During the test there was a reduction of HRV. The volunteers presented a similar profile in the response to the Modified Wingate Test, characterizing an increase of the RR intervals during the rest phase. This was followed by the decrease of these intervals in the submaximum and maximum phases and a gradual increase of the RR intervals in the active and passive phases, characterizing a decrease in the parasympathetic tonus, with the exception of one volunteer who maintained the decrease of the RR intervals in the active and passive recuperation phases.

4- The group presented a predominance of the parasympathetic system in the rest phase and, during the other protocol phases, a parasympathetic drop was observed according to the analysis of the RMSSD and PNN50 parameters.

## Figures and Tables

**Table 1:**
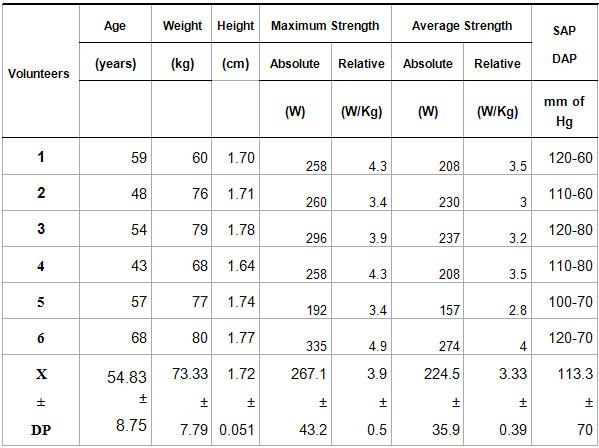
Anthropometric characteristics: weight (kg) and height (cm), age (years), values of maximum and average strength (W), maximum and average relative strength (W.Kg) and systolic and diastolic arterial pressure (mmHg), with their respective averages and standard deviations.

SAP - Systolic arterial pressure; DAP - Diastolic arterial pressure

**Table 2:**
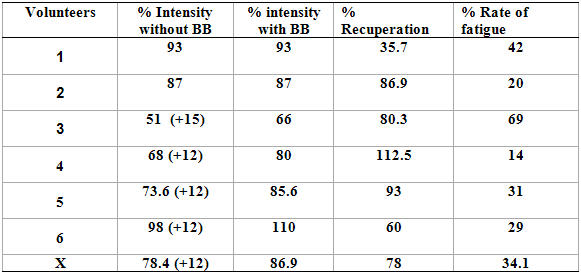
Volunteers' percentages of test intensity with and without beta- blockers, recuperation and rate of fatigue

**Table 3:**
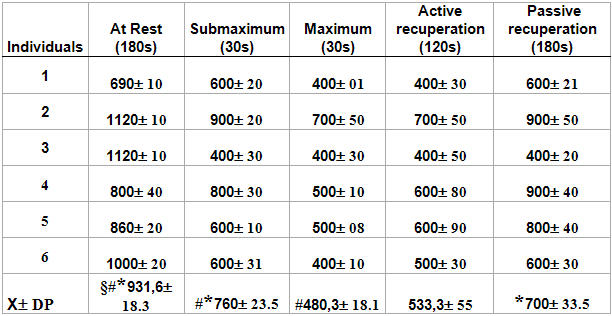
Volunteers' average values and standard deviations at the RR intervals (MNN and SDNN in milliseconds during the different phases of the test.

p<0.01 in all phases of the rest protocol (rejection Ho) *p<0.05 in relation to the maximum phase in the MNN #p<0.05 in relation to the active recuperation phase in the SDNN §p<0.05 in relation to the passive recuperation phase on the SDNN

**Table 4:**
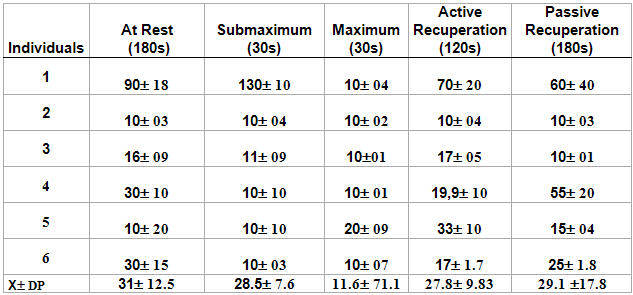
Volunteers' average values and standard deviations according to the RMSSD parameter (in milliseconds) in the different phases of the test

p>0.05 in all phases

**Table 5:**
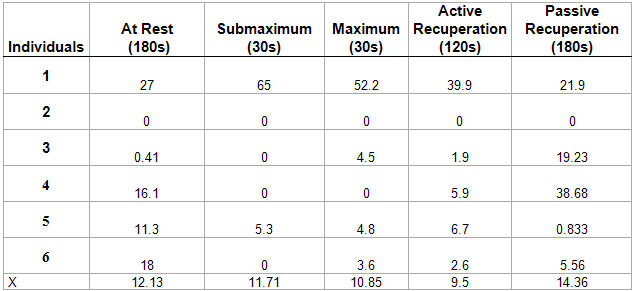
Average values and standard deviations of the PNN50 (%) in the different phases of the test

p>0.05 in all phases
